# Anti-Inflammatory Activity of Glabralactone, a Coumarin Compound from *Angelica sinensis*, via Suppression of TRIF-Dependent IRF-3 Signaling and NF-*κ*B Pathways

**DOI:** 10.1155/2022/5985255

**Published:** 2022-05-09

**Authors:** Tae Jun Choi, Jayoung Song, Hyen Joo Park, Sam Sik Kang, Sang Kook Lee

**Affiliations:** College of Pharmacy, Natural Products Research Institute, Seoul National University, 1 Gwanak-ro, Gwanak-gu, Seoul 08826, Republic of Korea

## Abstract

The dried root of *Angelica sinensis* (*A. sinensis*) has been widely used in Chinese traditional medicine for various diseases such as inflammation, osteoarthritis, infections, mild anemia, fatigue, and high blood pressure. Searching for the secondary metabolites of *A. sinensis* has been mainly conducted. However, the bioactivity of coumarins in the plant remains unexplored. Therefore, this study was designed to evaluate the anti-inflammatory activity of glabralactone, a coumarin compound from *A. sinensis*, using *in vitro* and *in vivo* models, and to elucidate the underlying molecular mechanisms of action. Glabralactone effectively inhibited nitric oxide production in lipopolysaccharide- (LPS-) stimulated RAW264.7 macrophage cells. The downregulation of LPS-induced mRNA and protein expression of iNOS, TNF-*α*, IL-1*β*, and miR-155 was found by glabralactone. The activation of NF-*κ*B and TRIF-dependent IRF-3 pathway was also effectively suppressed by glabralactone in LPS-stimulated macrophages. Glabralactone (5 and 10 mg/kg) exhibited an *in vivo* anti-inflammatory activity with the reduction of paw edema volume in carrageenan-induced rat model, and the expressions of iNOS and IL-1*β* proteins were suppressed by glabralactone in the paw soft tissues of the animal model. Taken together, glabralactone exhibited an anti-inflammatory activity in *in vitro* and *in vivo* models. These findings reveal that glabralactone might be one of the potential components for the anti-inflammatory activity of *A. sinensis* and may be prioritized in the development of a chemotherapeutic agent for the treatment of inflammatory diseases.

## 1. Introduction

Inflammation serves as a protective response to injurious stimuli. Therefore, an acute inflammatory process is considered a crucial response for a host to maintain homeostasis. However, a pathological condition is developed when the inflammation is heightened and prolonged, and it is responsible for many pathophysiological diseases, such as rheumatoid arthritis, Alzheimer's disease, asthma, and cancer [1, 2]. Macrophages, which play a central role in inflammation and host defense mechanisms, produce several proinflammatory mediators, including nitric oxide (NO) and various proinflammatory cytokines such as tumor necrosis factor-*α* (TNF-*α*), interferon-*γ* (IFN-*γ*), and interleukins [3]. When a lipopolysaccharide (LPS) interacts with a dimerized receptor, myeloid differentiation protein-2 (MD-2), and toll-like receptor 4 (TLR4), the inflammation response is activated via macrophages [4]. The following signal transductions depend on different adapters and are broadly classified into myeloid differentiation factor 88- (MyD88-) dependent pathway and TIR-domain-containing adaptor protein inducing IFN-*β*- (TRIF-) dependent pathway.

Nuclear factor-*κ*B (NF-*κ*B) is known to be a crucial transcriptional factor regulating inducible nitric oxide synthase (iNOS) gene expression. In unstimulated cells, NF-*κ*B exists as inactive heterodimers (p65 and p50) bound to I*κ*B-*α*, the inhibitory protein of NF-*κ*B, in the cytosol. When stimulated by proinflammatory signals, I*κ*B kinase (IKK) phosphorylates I*κ*B-*α* which is then inactivated through ubiquitin-mediated degradation. These events lead to the translocation of NF-*κ*B into the nucleus and subsequently regulate its target gene transcription [5]. In general, the MyD88-dependent pathway involves an early response that leads to NF-*κ*B activation. In contrast, the TRIF-dependent pathway results in the late activation of NF-*κ*B and interferon- (IFN-) regulatory factor 3 (IRF3) [6, 7].

Both of the signaling pathways lead to the induction of inflammation-related cytokines, chemokines, and other transcription factors [7]. In MyD88-dependent signaling pathway, the mitogen-activated protein kinases (MAPKs) are activated and eventually, AP-1, a transcription factor regulating inflammation-related gene expression, is activated. In the TRIF-dependent signaling pathway, signal transducer and activator of transcription 1 (STAT1) and IFN regulatory factor (IRF) proteins are activated, which is correlated with the expression of IFN-*β*. It is also known that the activation of STAT1 by LPS plays critical roles in expressing a subset of inflammation factors such as NO and prostaglandins [8].

A variety of natural product-derived compounds have shown anti-inflammatory properties with inhibitory activities of inducible nitric oxide synthase (iNOS) in LPS-activated macrophage cells [9–11]. *Angelica sinensis* (Oliv.) Diels (Apiaceae) is a medicinal plant widely found in Korea and mainland China. It has been traditionally used for gynecological conditions, fatigue, anemia, hypertension, inflammation, osteoarthritis, and migraine headache [12]. Although bioactivities of several compounds from *A. sinensis* are quite well discovered, the bioactivity of coumarins remains relatively unexplored.

**Figure 1:**
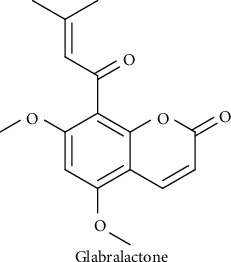
Chemical structure of glabralactone.

In our continuous efforts to discover anti-inflammatory agents from natural sources, we extended our research to evaluate the effect of glabralactone (Figure 1, Supplementary Material (available here)), a coumarin compound from *A. sinensis*, on inflammatory responses. Here, the anti-inflammatory activity and its underlying molecular mechanisms of glabralactone were investigated in *in vitro* and *in vivo* animal models.

## 2. Material and Methods

### 2.1. Chemicals

Dulbecco's modified Eagle's medium (DMEM), fetal bovine serum (FBS), sodium pyruvate, L-glutamine, antibiotic−antimycotic solution, and trypsin-EDTA were purchased from Invitrogen Co. (Grand Island, NY, USA). Goat antirabbit IgG-HRP, goat anti-mouse IgG-horseradish peroxidase (HRP), mouse anti-goat IgG-HRP, iNOS, I*κ*B-*α*, p-I*κ*B-*α*, p50, p65, IL-1*β*, ERK1/2, p-ERK1/2, and *β*-actin mouse monoclonal antibodies were purchased from Santa Cruz Biotechnology, Inc. (Santa Cruz, CA, USA). Antibodies against TNF-*α*, SHIP1, PTEN, p38, p-p38, SAPK/JNK, p-SAPK/JNK, IRF3, p-IRF3, STAT1, p-STAT1, p-JAK1 rabbit monoclonal antibodies, and JAK1 mouse monoclonal antibody were purchased from Cell Signaling Technology (Beverly, MA, USA). AMV reverse transcriptase, dNTP mixture, random primer, RNasin, and Taq polymerase were purchased from Promega (Madison, WI, USA). Lipopolysaccharide (E. coli O111: B4), polyinosinic-polycytidylic acid (poly (I:C)), 3-(4,5-dimethylthiazol-2-yl)-2,5-diphenyltetrazolium bromide (MTT), dimethyl sulfoxide (DMSO), and other chemicals were purchased from Sigma-Aldrich (St. Louis, MO, USA), unless otherwise indicated. Glabralactone (purity > 96% by HPLC analysis) was isolated from an extract of the roots of *Angelica sinensis* (see Supplementary Material Information).

### 2.2. Cell Culture

A RAW 264.7 murine macrophage cell line was obtained from the American Type Culture Collection (ATCC, Rockville, MD, USA). Cells were cultured in DMEM supplemented with 10% heat-inactivated FBS, 100 units/mL penicillin, 100 *μ*g/mL streptomycin, and 0.25 *μ*g/mL amphotericin B. Cells were incubated in a humidified chamber at 37°C with 5% CO_2_ atmosphere.

### 2.3. Nitrite Assay

The effect of test compounds on the production of nitric oxide (NO) in the culture medium was determined as described previously [10].

### 2.4. MTT Cell Viability Assay

Following NO assessment, MTT solution was added to each well to make a final concentration of 500 *μ*g/mL. After 4 h incubation at 37°C, medium was discarded, and 1 mL of dimethyl sulfoxide was added to dissolve the remaining formazan. 100 *μ*L of the dissolved sample was transferred into a 96-well plate, and the absorbance was measured at 570 nm. The percent survival was determined by comparison with a control group.

### 2.5. Western Blotting

Cultured cells were collected in lysis buffer containing 50 mM Tris-HCl (pH 7.4), 150 mM NaCl, 10 mM EDTA, 0.5% NP-40, Complete™ protease inhibitor cocktail (Roche Applied Science, Penzberg, Germany), and PhosSTOP™ phosphatase inhibitor cocktail (Roche). After 10 min of centrifugation (15,000 rpm), supernatant containing the protein was collected. Protein concentration was measured by the bicinchoninic acid method and subjected to immunoblotting using a standard protocol. The blots were visualized using enhanced chemiluminescence (ECL) solution (Intron, Daejeon, Korea) on LAS-4000 Imager (GE Healthcare, Little Chalfont, UK) [13].

### 2.6. RNA Extraction and Real-Time Reverse Transcriptase-Polymerase Chain Reaction (Real-Time RT-PCR)

RAW 264.7 cells were stimulated with LPS (final concentration 1 *μ*g/mL) in the presence or absence of the test compound for 5 h. Total cellular RNA was extracted using TRI reagent (Sigma-Aldrich). From each sample, 1 *μ*g of total RNA was reverse-transcribed using a Reverse Transcription System (Promega) according to the manufacturer's instruction. The following primers were used for real-time RT-PCR: mouse IFN-*β* F5′-CATTTCCGAATGTTCGTCCT-3′; mouse IFN-*β* R5′-CACAGCCCTCTCCATCAACTA-3′; mouse TNF-*α* F5′-TTGAGATCCATGCCGTTG-3′; mouse TNF-*α* R5′-CTGTAGCCCACGTCGTAGC-3′; mouse IL-1*β* F5′ - AGCTGGATGCTCTCATCAGG-3′; mouse IL-1*β* R5′-AGTTGACGGACCCCAAAAG-3′; mouse *β*-actin F5′-ACCAGAGGCATACAGGGACA-3′; and mouse *β*-actin R5′-CTAAGGCCAACCGTGAAAAG-3′. Gene-specific primers were synthesized by Bioneer (Daejeon, Korea). Real-time PCR was carried out as described previously [10].

### 2.7. Reporter Gene (SEAP, Secreted Embryonic Alkaline Phosphatase) Assay

To determine the effect of glabralactone on the activation of NF-*κ*B, a reporter gene assay was performed as described previously with some modifications [10, 14]. Human HaCaT transfectant was established by stable transfection with pNF-kB-binding site-SEAP-NPT plasmid [14]. The cells were cultured in DMEM supplemented with 10% heat-inactivated FBS, 100 units/mL penicillin, 100 *μ*g/mL streptomycin, and 0.25 *μ*g/mL amphotericin B and incubated in a humidified chamber at 37°C with 5% CO_2_ atmosphere.

The cells were treated with test compound for 2 h and then further stimulated with LPS for an additional 16 h. The supernatants were heated at 65°C for 5 min and reacted with SEAP assay buffer (2 M diethanolamine, 1 mM MgCl_2_, and 500 *μ*M 4-methylumbelliferyl phosphate (MUP)) for 1 h. The fluorescence was measured in a 96-well plate fluorometer and normalized with protein concentration. Data are expressed as the proportion to vehicle-treated control cells without LPS.

### 2.8. Preparation of Nuclear Extracts

Nuclear extraction was performed using the nuclear extract kit (Active Motif North America, CA, USA) according to the manufacturer's instructions.

### 2.9. NF-*κ*B DNA-Binding Activity

Trans-AM NF-*κ*B transcription factor assay kit (Active Motif, Tokyo, Japan) was used to measure the NF-*κ*B DNA-binding activity according to the manufacturer's instructions [10].

### 2.10. MicroRNA-155 Determination

Quantitative RT-PCR analysis for microRNA-155 was carried out as described previously [10].

### 2.11. Animals

Sprague−Dawley (SD) rats (150−170 g) were used for *in vivo* studies (Central Laboratory Animal, Inc., Seoul, Korea). All animal experiments were conducted following the guidelines of the Seoul National University Institutional Animal Care and Use Committee (IACUC; permission number: SNU-130809-4).

### 2.12. Carrageenan-Induced Paw Edema

To assess anti-inflammatory activity of the test compounds, the carrageenan-induced hind paw edema model in rats was used [10, 15]. Glabralactone was dissolved in 0.5% CMC-Na. 30 min after intraperitoneal injection of test compounds, paw edema was induced by the subplantar injection of 1% carrageenan suspension in normal saline (100 *μ*L). The paw volume was measured at the indicated time after carrageenan injection using a plethysmometer (Ugo Basile, Comerio, Italy).

### 2.13. Determination of the Production of Proinflammatory Mediators in Inflamed Tissues

The whole-cell extract from inflamed tissues was obtained using a nuclear extract kit (Active Motif, Carlsbad, CA, USA) according to the manufacturer's instructions. Protein levels of proinflammatory mediators were determined by Western blotting.

### 2.14. Statistical Analysis

Each experiment was performed in triplicate. Results are presented as the means ± SD. The statistical significance was assessed using a one-way analysis of variation (ANOVA) coupled with Dunnett's *t*-test; *p* value < 0.05 was considered statistically significant.

## 3. Results

### 3.1. Effect of Glabralactone on the Formation of Nitrite in LPS-Induced RAW 264.7 Cells

**Figure 2:**
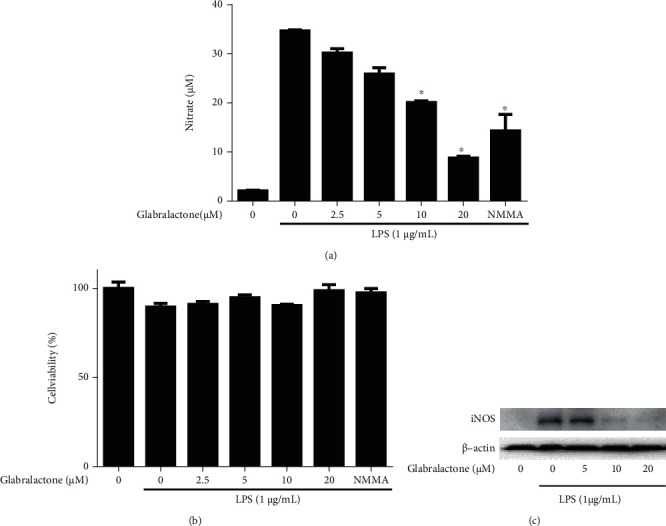
Inhibitory effects of glabralactone on NO production and iNOS expression in LPS-stimulated RAW 264.7 cells. (a) RAW 264.7 cells were stimulated with LPS (1 *μ*g/mL) in the presence or absence of glabralactone. After 20 h, the nitrite concentrations were analyzed in the cultured media. The values are expressed as the mean ± SD. ^∗^*p* < 0.05 was considered statistically significant. (b) Cell viability was determined using MTT assay. All experiments were performed in triplicate. The data are expressed as mean ± SD. (c) The regulation of iNOS protein expression by glabralactone was determined. Cells were treated with LPS (1 *μ*g/mL) and glabralactone (0–20 *μ*M) for 16 h. After incubation, Western blotting was performed as described in Materials and Methods. Data are representative of three separate experiments. *β*-Actin was used as an internal standard. Compounds were dissolved in 100% DMSO, and the final concentration of DMSO was adjusted to 0.2% (a), 0.5% (b), and 0.1% (c).

To evaluate the effect of glabralactone on the inhibition of NO production, murine macrophage RAW 264.7 cells were stimulated with LPS, and the amount of nitrite, a stable metabolite of NO, in the medium was measured. As shown in Figure 2(a), the NO production by LPS (1 *μ*g/mL) treatment was prominently induced from the basal level of 2.1 ± 0.1 *μ*M to 34.7 ± 0.2 *μ*M for 20 h incubation. When the cells were pretreated with glabralactone (0–20 *μ*M) for 30* *min prior to LPS stimulation, NO production was inhibited concentration dependently with an IC_50_ value of 11.6 *μ*M. At the highest concentration (20 *μ*M) of glabralactone, NO production was inhibited by approximately 80%. N*ω*-monomethyl-L-arginine acetate (L-NMMA), a nonselective inhibitor of NOS, was used as a positive control and exhibited the inhibition of NO production (approximately 60% inhibition at 50 *μ*M). When the effect of glabralactone on cell viability was determined using the MTT assay, no significant cytotoxicity was observed at a test concentration of up to 20 *μ*M of glabralactone (>89% cell survival). These data indicate that glabralactone effectively inhibits NO production without cytotoxic activity (Figure 2(b)).

To further investigate the mechanism of action of glabralactone, the protein expression of iNOS was determined using Western blotting. When RAW 264.7 cells were treated with LPS (1 *μ*g/mL), iNOS protein level was significantly upregulated, but the presence of glabralactone effectively suppressed the iNOS protein expression in a concentration-dependent manner (Figure 2(c)). These data suggest that the inhibitory activity of glabralactone on NO production was correlated with the suppression of iNOS protein expression in LPS-stimulated RAW264.7 cells.

### 3.2. Effect of Glabralactone on LPS-Induced NF-*κ*B Activation

**Figure 3:**
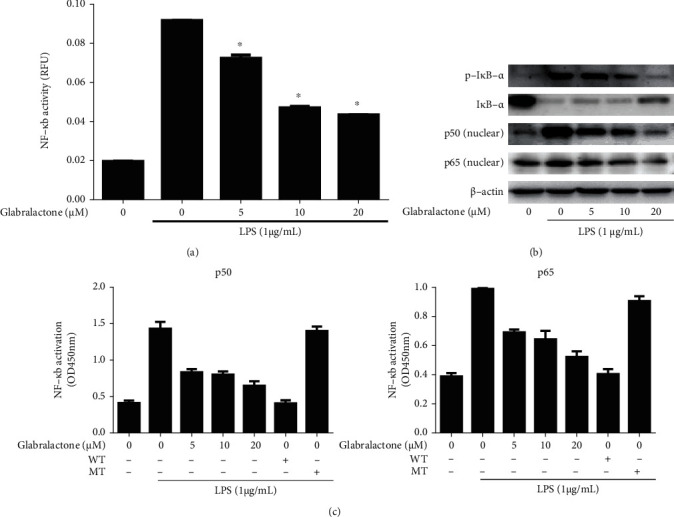
Effect of glabralactone on LPS-stimulated NF-*κ*B transcriptional activity. (a) SEAP-RAW cells were stimulated with LPS (1 *μ*g/mL). After 16 h of glabralactone treatment, the supernatants were analyzed. The values are expressed as the mean ± SD. ^∗^*p* < 0.05 was considered statistically significant. (b) Effects of glabralactone on the degradation of I*κ*B-*α* and on the expression of NF-*κ*B subunits in the nucleus. RAW 264.7 cells were pretreated with glabralactone for 30 min and stimulated with LPS for 30 min. The extracted proteins were analyzed by Western blotting. Data are representative of three separate experiments. *β*-Actin was used as an internal standard. (c) Effects of glabralactone on the LPS-stimulated NF-*κ*B DNA-binding activity. Cells were stimulated with LPS (1 *μ*g/mL) for 1.5 h in the presence of glabralactone, and the isolated nuclear extracts were analyzed for NF-*κ*B DNA-binding activity. The values are expressed as the means ± SD. (left for p50, right for p65). Glabralactone was dissolved in 100% DMSO, and the final concentration of DMSO was adjusted to 0.1%.

To further investigate whether the transcription factor NF-*κ*B is a crucial target for the effect of glabralactone in LPS-stimulated RAW 264.7 cells, a reporter gene assay for NF-*κ*B transcriptional activity (SEAP assay) was performed. When the cells were stimulated with LPS (1 *μ*g/mL) for 16 h, the NF-*κ*B transcriptional activity was increased with a 4.7-fold. However, the treatment of glabralactone effectively inhibited the NF-*κ*B transcriptional activity in a concentration-dependent manner in LPS-stimulated RAW264.7 cells (Figure 3(a)).

Further study was designed to elucidate the effect of glabralactone on the activation of NF-*κ*B in LPS-stimulated RAW264.7 cells. The LPS treatment (1 *μ*g/mL) for 30 min exhibited the significant downregulation of I*κ*B-*α* and the upregulation of p-I*κ*B-*α* (degradation form of I*κ*B-*α*). However, the pretreatment of glabralactone for 30 min prior to LPS stimulation suppressed the degradation of I*κ*B-*α* in the cytosol and the translocation of the NF-*κ*B subunits (p65 and p50) in the nucleus (Figure 3(b)).

To determine the effect of glabralactone on the DNA binding activity of NF-*κ*B in the nucleus, RAW264.7 cells were treated with LPS (1 *μ*g/mL) in the presence of glabralactone for 1.5 h, and then, the nuclear extracts were analyzed by Trans-AM NF-*κ*B binding assay (Active Motif). The enhanced DNA-binding activity of NF-*κ*B subunits (p50 and p65) by LPS stimulation in the nucleus was effectively inhibited by glabralactone in a concentration-dependent manner. The competition experiments with the addition of excess unlabeled wild-type (WT) and mutated (MT) oligonucleotides (p50 or p65) confirmed that only the addition of WT oligonucleotides completely blocks the DNA-binding activity of NF-*κ*B in the nucleus. These data indicate that the inhibition of DNA-binding activity of NF-*κ*B by glabralactone is specific to the subunits in the nucleus.

### 3.3. Effects of Glabralactone on Proinflammatory Cytokine and miRNA-155 Expression

**Figure 4:**
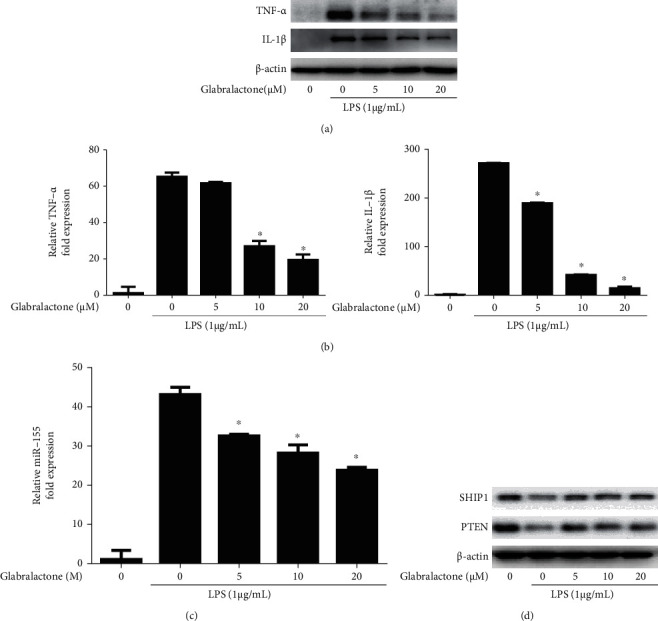
Effect of glabralactone on LPS-induced proinflammatory cytokine and miR-155 expressions. The proteins (a) and mRNA (b) levels of TNF-*α* and IL-1*β* were determined by Western blotting and qRT-PCR, respectively. Cells were stimulated with LPS (1 *μ*g/mL) in the presence of glabralactone for 4 h and was further analyzed as described in Materials and Methods. Data are representative of three separate experiments. *β*-Actin was used as an internal standard. (c) Effect of glabralactone on the expression of miR-155 in LPS-stimulated RAW264.7 cells. The cells were stimulated with LPS in the presence of glabralactone for 8 h. The expression of miRNA-155 was determined by qRT-PCR as described in Materials and Methods. The values are expressed as the means ± SD. (b, c) ^∗^*p* < 0.05 was considered statistically significant. (d) Effect of glabralactone on the expression of SHIP1 and PTEN in macrophage cells. Cells were treated with glabralactone for 30 min and then stimulated with LPS for 30 min. Proteins were analyzed by Western blotting. Data are representative of three separate experiments. *β*-Actin was used as an internal standard. Glabralactone was dissolved in 100% DMSO, and the final concentration of DMSO was adjusted to 0.1%.

To further elucidate whether the anti-inflammatory effect of glabralactone is associated with the regulation of the proinflammatory cytokine expression, primarily, the expressions of TNF-*α* and IL-1*β* were determined in LPS-stimulated RAW264.7 cells. The upregulated expressions of TNF-*α* and IL-1*β* by LPS treatment for 4 h were effectively suppressed by the treatment of glabralactone in the protein (Figure 4(a)) and mRNA levels (Figure 4(b)) in LPS-stimulated RAW264.7 cells.

It is well known that miRNA-155 (miR-155) plays an essential role in an inflammatory response. It was also demonstrated that proinflammatory cytokines, such as TNF-*α* and IL-1*β*, are positively regulated by miR-155 [16]. On this line, the effect of glabralactone on the expression of miR-155 was determined in LPS-stimulated RAW264.7 cells. When the cells were stimulated with LPS (1 *μ*g/mL) for 8 h, the expression of miR-155 was increased with 43.0-fold higher than that of unstimulated cells. However, the upregulated expression of miR-155 was significantly suppressed by glabralactone in a concentration-dependent manner in LPS-stimulated RAW264.7 cells (Figure 4(c)). An additional study revealed that the expressions of SHIP1 and PTEN, the negatively regulated target proteins of miR-155, were affected by the treatment of glabralactone (Figure 4(d)), indicating that the anti-inflammatory activity of glabralactone is in part correlated with the downregulation of miR-155 expression in RAW264.7 cells.

### 3.4. Effect of Glabralactone on TRIF-Dependent TLR Pathway

**Figure 5:**
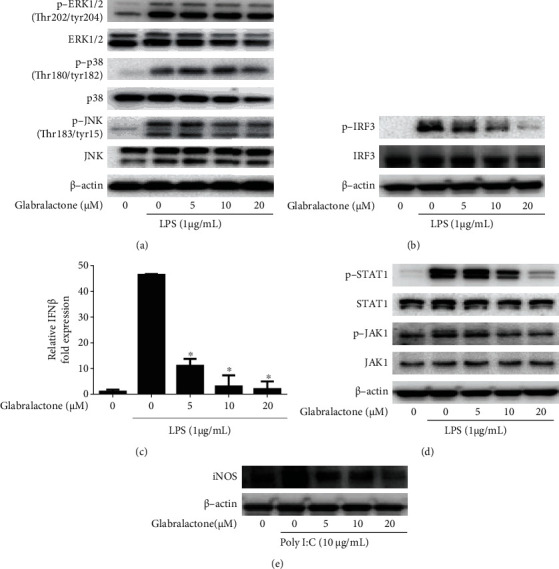
Effect of glabralactone on the TRIF-dependent TLR signaling pathway. (a) Effect of glabralactone on the protein expression of MAPK signaling in LPS-stimulated RAW 264.7 cells. Cells were pretreated with glabralactone for 30 min and then stimulated with LPS (1 *μ*g/mL) for an additional 30 min. Proteins were analyzed by Western blotting. (b) Effect of glabralactone on the protein expression of IRF3 in LPS-stimulated RAW 264.7 cells. Cells were pretreated with glabralactone for 30 min and then stimulated with LPS (1 *μ*g/mL) for 3 h. After incubation, total proteins were extracted, and IRF3 and p-IRF3 protein expressions were analyzed by Western blotting. (c) Effect of glabralactone on the mRNA expression of IFN-*β* in LPS-stimulated RAW 264.7 cells. Cells were pretreated with glabralactone for 30 min and then stimulated with LPS (1 *μ*g/mL) for 4 h. The mRNA expression of IFN-*β* was analyzed by using reverse transcription and real-time PCR. ^∗^*p* < 0.05. (d) Effect of glabralactone on the JAK/STAT pathway protein expression in LPS-stimulated RAW 264.7 cells. Cells were pretreated with glabralactone for 30 min and then stimulated with LPS (1 *μ*g/mL) for 3 h. After incubation, total proteins were extracted, and protein level was analyzed by Western blotting. (e) Effect of glabralactone on poly (I:C)-induced iNOS expression. RAW 264.7 cells were pretreated with glabralactone for 30 min and then stimulated with poly I:C (10 *μ*g/mL) for 12 h. After incubation, total proteins were extracted, and iNOS protein level was analyzed by Western blotting. *β*-Actin was used as an internal standard. Glabralactone was dissolved in 100% DMSO, and the final concentration of DMSO was adjusted to 0.1%.

To investigate whether glabralactone is able to affect the MyD88-dependent TLR signaling pathway, the expressions of the MAPK family, such as JNK, ERK, and p38, were examined by Western blotting. As shown in Figure 5(a), the activation (phosphorylated form) of ERK, p38, and JNK was not much affected by glabralactone treatment in LPS-stimulated RAW264.7 cells.

Next, to further investigate whether glabralactone is able to regulate the TRIF-dependent TLR signaling pathway, the effect of glabralactone on the activation of IRF3 was determined in LPS-stimulated RAW264.7 cells. As shown in Figure 5(b), LPS treatment (1 *μ*g/mL) for 3 h significantly enhanced the expression of p-IRF3 (activation form of IRF3), but the pretreatment of glabralactone for 30 min prior to LPS stimulation effectively downregulated the expression of p-IRF3 in a concentration-dependent manner. Subsequent study was designed to evaluate the effect of glabralactone on the transcriptional target gene expression of IRF3. The mRNA expression of IFN*β*, a transcriptional target gene of IRF3, was significantly upregulated by LPS treatment (1 *μ*g/mL) for 4 h, but the pretreatment of glabralactone for 30 min prior to LPS stimulation also effectively suppressed the mRNA expression of IFN*β* in LPS-stimulated RAW264.7 cells (Figure 5(c)). In addition, since the JAK-STAT pathway is downstream of IFN*β*, the effect of glabralactone on the activation of JAK1 and STAT1 was examined by Western blotting in LPS-stimulated RAW264.7 cells. As a result, the enhanced activation of STAT1 and JAK1 (phosphorylation forms) protein expression by LPS treatment for 3 h was effectively downregulated in the presence of glabralactone (Figure 5(d)). To further confirm that the suppression of iNOS expression by glabralactone is mediated by TRIF-dependent pathway, polyinosinic–polycytidylic acid (poly (I:C)) was used to induce iNOS expression in RAW264.7 cells. The treatment of poly (I:C) for 12 h significantly induced the iNOS protein expression, and the pretreatment of glabralactone for 30 min prior to LPS stimulation effectively downregulated the iNOS expression in a concentration-dependent manner (Figure 5(e)). These findings suggest that the suppressive effect of iNOS expression is in part associated with the regulation of TRIF-dependent TLR pathway.

### 3.5. *In Vivo* Anti-Inflammatory Activity of Glabralactone

**Figure 6:**
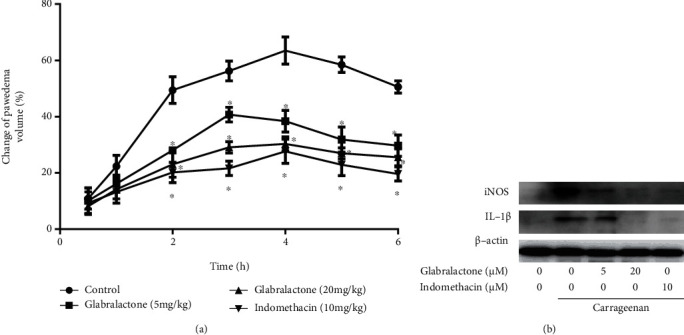
*In vivo* anti-inflammatory activity of glabralactone. (a) Inhibitory effect of glabralactone on the carrageenan-induced paw edema. Glabralactone was orally administered 30 min before subplantar injection of carrageenan. The paw volume was measured at 0, 0.5, 1, 2, 3, 4, 5, and 6 h after injection using a plethysmometer. ^∗^*p* < 0.05. (b) Effect of glabralactone on the proinflammatory protein expression in the carrageenan-induced paw edema model. The soft paw tissues were amputated and homogenized using a nuclear extract kit (Active Motif, Carlsbad, CA). The expressions of iNOS and IL-1*β* proteins were examined by Western blotting. *β*-Actin was used as an internal standard.

A carrageenan-induced rat paw edema model was used to evaluate the *in vivo* anti-inflammatory activity of glabralactone. Paw edema was induced by injecting 100 *μ*L of 1% solution of carrageenan in distilled water subplantarly, and the volume of paw edema was monitored for 6 h. The volume of paw edema was increased continuously and peaked at 4 h after carrageenan treatment. As shown in Figure 6(a), when glabralactone was orally treated, the paw edema volume was significantly decreased compared to the vehicle-treated control groups. The inhibition rate was 39.58% and 52.24% at doses of 5 and 20 mg/kg, respectively, after 4 h treatment of glabralactone. Indomethacin (20 mg/kg) was used as a reference compound, and under the same experimental conditions, the inhibition rate was 56.41%.

The production of proinflammatory mediators after treatment of glabralactone in inflamed paw tissue was also assessed by Western blotting (Figure 6(b)). After treatment of carrageenan for 6 h, the protein expressions of the proinflammatory mediators, iNOS and IL-1*β*, in paw tissues were upregulated compared to the vehicle-treated control groups. However, the expression of these proinflammatory mediators was significantly suppressed by the treatment of glabralactone (20 mg/kg). These findings suggest that glabralactone exhibits the *in vivo* anti-inflammatory activity in acute inflammation animal model.

## 4. Discussion

Natural products have been playing an important role in drug discovery and development program. Indeed, over 50% of all approved small molecule drugs are based on diverse natural product-originated compounds [17]. *Angelica sinensis* (*A. sinensis*), a medicinal plant that is mainly distributed in Korea and mainland China, has been traditionally used for various human health including inflammatory diseases. *A. sinensis* is also commonly known as female ginseng because of benefit to women's health [12]. Diverse classes of compounds such as essential oils, coumarins, phthalides, polysaccharides, and polyacetylenes have been isolated from *A. sinensis* [18]. Although bioactivities of isolated compounds such as ferulic acid, Z-ligustilide, and n-butylidenephthalide from *A. sinensis* have been reported [19], the anti-inflammatory activity of isolated coumarin compounds from *A. sinensis* remains to be explored. In our continuous efforts to search for anti-inflammatory agents from natural sources and based on the use of inflammation diseases of the plant, the present study was conducted to evaluate the anti-inflammatory activity of glabralactone, a coumarin compound from *A. sinensis*, in inflammatory responses.

The overproduction of nitric oxide (NO) and inducible nitric oxide synthase (iNOS) in activated macrophages is highly correlated with inflammatory responses [20]. Therefore, the regulation of the production of NO and the expression of iNOS is largely considered as a useful target in the screening of anti-inflammatory agents. In the present study, we employed the *in vitro* assay system with lipopolysaccharide- (LPS-) stimulated mouse macrophage RAW264.7 cells for activation of macrophages. LPS, an endotoxin of Gram-negative bacteria, is a well-known activator of macrophages in inflammatory response [21]. Glabralactone was found to significantly inhibit the production of NO in LPS-stimulated macrophage cells without cytotoxicity. Since the overproduction of NO is in part associated with the overexpression of iNOS in activated macrophages, the underlying molecular mechanism mediated by glabralactone was elucidated in LPS-stimulated macrophage cells. Western blot analysis revealed that glabralactone also suppressed the LPS-stimulated overexpression of iNOS protein levels in activated macrophage cells. These findings suggest that the inhibition of NO production by glabralactone is associated with the suppression of iNOS protein expression. Further study was designed to elucidate the involvement of NF-*κ*B as a crucial transcriptional factor in the regulation of iNOS expression by glabralactone. The enhanced NF-*κ*B transcriptional activity with LPS-stimulation was effectively inhibited by the treatment of glabralactone analyzed by SEAP assay, and subsequently, these effects were associated with the suppression of the degradation of I*κ*B-*α* in cytosol and the translocation of NF-*κ*B subunits (p50 and p65) into the nucleus. The suppression of NF-*κ*B translocation into the nucleus by glabralactone was also confirmed by the analysis of NF-*κ*B-DNA binding activity along with competition experiments using wild type or mutated type of oligonucleotides of NF-*κ*B subunits in LPS-stimulated macrophage cells. These data indicate that the anti-inflammatory activity of glabralactone is in part associated with the suppression of the activation of NF-*κ*B in activated macrophages.

Accumulating evidences reveal that proinflammatory cytokines such as TNF-*α* and IL-1*β* are overexpressed in inflammatory responses, and NF-*κ*B plays a critical role in the expression of these proinflammatory mediators [22]. Since glabralactone effectively suppressed the NF-*κ*B activation, the expressions of the proinflammatory cytokines were determined in LPS-stimulated macrophage cells. Glabralactone downregulated the expressions of TNF-*α* and IL-1*β* at the transcriptional and translational levels, indicating that the anti-inflammatory activity of glabralactone is also associated with the suppression of proinflammatory cytokines in activated macrophage cells.

The signal transduction pathway in inflammatory responses is generally classified into Myd88-dependent and TRIF-dependent signaling pathways. In Myd88-dependent signaling, the activation of MAPKs is normally found, but in TRIF-dependent signaling, the activation of interferon- (IFN-) regulatory factor 3 (IRF3) and its downstream signaling molecules is mediated in inflammatory responses [6]. In the present study, glabralactone was found to more effectively suppress the activation of TRIF-dependent IRF3 signaling pathways compared to that of the MyD88-dependent pathway in LPS-stimulated macrophage cells. Glabralactone significantly suppressed the activation (phosphorylation) of IRF3 protein and IFN*β* mRNA expression and subsequently suppressed the activation of its downstream signaling molecules such as the phosphorylation of JAK1 and STAT1. The association of the TRIF-mediated signaling pathway in the anti-inflammatory activity of glabralactone was further confirmed by using poly (I:C). Poly (I:C), a synthetic analog of double-stranded RNA (dsRNA), is known to induce the production of cytokines such as IFN*β*. Poly (I:C) is able to activate TRL3 which precisely mediates the TRIF-dependent pathway. Therefore, the activation of the TLR3-TRIF pathway causes the production of inflammatory cytokines and chemokines [23]. Glabralactone was found to effectively suppress poly (I:C)-induced iNOS protein expression. These findings suggest that the suppressive effect of iNOS expression is in part associated with the regulation of the TRIF-dependent TLR pathway. Taken together, these data indicate that glabralactone might affect the TRIF-mediated signaling pathway.

The *in vivo* anti-inflammatory activity of glabralactone was also evaluated using a carrageenan-induced rat paw edema model. Glabralactone significantly reduced the volume of carrageenan-induced paw edema. Tissue analysis also suggested that the anti-inflammatory activity of glabralactone was in part associated with the suppression of the production of proinflammatory mediators, iNOS and IL-1*β*, in inflamed paw edema tissues in rat models.

A variety of natural product-derived compounds such as handelin, curcumin, isoliquiritigenin, auranofin, luteolin, (–)-epigallocatechin-3-gallate, 6-shogaol, and pinosylvin have exhibited the anti-inflammatory activity with various molecular mechanisms [9, 10, 24–29]. This study is also considered to be meaningful because it gives a scientific relevance for the elucidation of isolated compounds based on the traditional use of *A. sinensis* in inflammatory diseases.

## 5. Conclusions

The present study provides the anti-inflammatory activity of glabralactone in both *in vitro* cell culture and *in vivo* acute paw edema rat models. A plausible underlying molecular mechanism of action for the anti-inflammatory activity of glabralactone involves the suppression of proinflammatory mediators and cytokine production via the modulation of the TRIF-dependent pathway. These findings suggest that glabralactone may serve as a promising candidate for further development of anti-inflammatory agents from natural products.

## Data Availability

Data is available on request.

## References

[1] Nathan C., Ding A. (2010). Nonresolving inflammation. *Cell*.

[2] Tabas I., Glass C. K. (2013). Anti-inflammatory therapy in chronic disease: challenges and opportunities. *Science*.

[3] Ross E. A., Devitt A., Johnson J. R. (2021). Macrophages: the good, the bad, and the gluttony. *Frontiers in Immunology*.

[4] Pålsson-McDermott E. M., O'Neill L. A. (2004). Signal transduction by the lipopolysaccharide receptor, Toll-like receptor-4. *Immunology*.

[5] Barnabei L., Laplantine E., Mbongo W., Rieux-Laucat F., Weil R. (2021). NF-*κ*B: at the borders of autoimmunity and inflammation. *Frontiers in Immunology*.

[6] Akira S., Takeda K. (2004). Toll-like receptor signalling. *Nature Reviews. Immunology*.

[7] Duan T., du Y., Xing C., Wang H. Y., Wang R. F. (2022). Toll-like receptor signaling and its role in cell-mediated immunity. *Frontiers in Immunology*.

[8] Luo L., Lucas R. M., Liu L., Stow J. L. (2020). Signalling, sorting and scaffolding adaptors for Toll-like receptors. *Journal of Cell Science*.

[9] Park E. J., Min H. Y., Chung H. J., Ahn Y. H., Pyee J. H., Lee S. K. (2011). Pinosylvin suppresses LPS-stimulated inducible nitric oxide synthase expression via the MyD88-independent, but TRIF-dependent downregulation of IRF-3 signaling pathway in mouse macrophage cells. *Cellular Physiology and Biochemistry*.

[10] Pyee Y., Chung H. J., Choi T. J. (2014). Suppression of inflammatory responses by handelin, a guaianolide dimer from Chrysanthemum boreale, via downregulation of NF-*κ*B signaling and pro-inflammatory cytokine production. *Journal of Natural Products*.

[11] Jin Lee E., Chung H. J., Pyee Y. (2014). Suppression of inducible nitric oxide synthase expression by nyasol and broussonin A, two phenolic compounds from *Anemarrhena asphodeloides*, through NF-*κ*B transcriptional regulation in vitro and in vivo. *Chemistry & Biodiversity*.

[12] Dinesh P., Rasool M., Watson R. R., Preedy V. R. (2019). Chapter 22 - herbal formulations and their bioactive components as dietary supplements for treating rheumatoid arthritis, in Bioactive Food as Dietary Interventions for Arthritis and Related Inflammatory Diseases.

[13] Kwon Y., Song J., Lee B. (2013). Design, synthesis, and evaluation of a water-soluble antofine analogue with high antiproliferative and antitumor activity. *Bioorganic & Medicinal Chemistry*.

[14] Moon K. Y., Hahn B. S., Lee J., Kim Y. S. (2001). A cell-based assay system for monitoring NF-*κ*B activity in human HaCaT transfectant cells. *Analytical Biochemistry*.

[15] Chung H. J., Lee J., Shin J. S. (2016). In vitro and in vivo anti-allergic and anti-inflammatory effects of eBV, a newly developed derivative of bee venom, through modulation of IRF3 signaling pathway in a carrageenan-induced edema model. *PLoS One*.

[16] Ceppi M., Pereira P. M., Dunand-Sauthier I. (2009). MicroRNA-155 modulates the interleukin-1 signaling pathway in activated human monocyte-derived dendritic cells. *Proceedings of the National Academy of Sciences of the United States of America*.

[17] Newman D. J., Cragg G. M. (2020). Natural products as sources of new drugs over the nearly four decades from 01/1981 to 09/2019. *Journal of Natural Products*.

[18] Yi L., Liang Y., Wu H., Yuan D. (2009). The analysis of Radix Angelicae Sinensis (Danggui). *Journal of Chromatography. A*.

[19] Chao W. W., Lin B. F. (2011). Bioactivities of major constituents isolated from Angelica sinensis (Danggui). *Chinese Medicine*.

[20] Patel R. P., McAndrew J., Sellak H. (1999). Biological aspects of reactive nitrogen species. *Biochimica et Biophysica Acta*.

[21] Sweet M. J., Hume D. A. (1996). Endotoxin signal transduction in macrophages. *Journal of Leukocyte Biology*.

[22] Stewart A. G., Beart P. M. (2016). Inflammation: maladies, models, mechanisms and molecules. *British Journal of Pharmacology*.

[23] Yamamoto M., Sato S., Hemmi H. (2003). Role of adaptor TRIF in the MyD88-independent toll-like receptor signaling pathway. *Science*.

[24] Youn H. S., Saitoh S. I., Miyake K., Hwang D. H. (2006). Inhibition of homodimerization of Toll-like receptor 4 by curcumin. *Biochemical Pharmacology*.

[25] Park S. J., Song H. Y., Youn H. S. (2009). Suppression of the TRIF-dependent signaling pathway of toll-like receptors by isoliquiritigenin in RAW264.7 macrophages. *Molecules and Cells*.

[26] Park S. J., Lee A. N., Youn H. S. (2010). TBK1-targeted suppression of TRIF-dependent signaling pathway of toll-like receptor 3 by auranofin. *Archives of Pharmacal Research*.

[27] Lee J. K., Kim S. Y., Kim Y. S., Lee W. H., Hwang D. H., Lee J. Y. (2009). Suppression of the TRIF-dependent signaling pathway of Toll-like receptors by luteolin. *Biochemical Pharmacology*.

[28] Park S. J., Lee M. Y., Son B. S., Youn H. S. (2009). TBK1-targeted suppression of TRIF-dependent signaling pathway of toll-like receptors by 6-shogaol, an active component of ginger. *Bioscience, Biotechnology, and Biochemistry*.

[29] Youn H. S., Lee J. Y., Saitoh S. I. (2006). Suppression of MyD88- and TRIF-dependent signaling pathways of toll-like receptor by (−)-epigallocatechin-3-gallate, a polyphenol component of green tea. *Biochemical Pharmacology*.

