# Supplementary material for: Anti-Inflammatory Activity of Glabralactone, a Coumarin Compound from Angelica sinensis, via Suppression of TRIF-Dependent IRF-3 Signaling and NF-κB Pathways
Source: Mediators Inflamm. 2022 May 9;2022:5985255. doi: 10.1155/2022/5985255 (PMC9110254; doi:10.1155/2022/5985255)

*Supplementary Material*

**Anti-inflammatory Activity of Glabralactone, a Coumarin Compound from *Angelica sinensis*, via Suppression of TRIF-dependent IRF-3 Signaling and NF-κB Pathways**

Tae Jun Choi^†^, Jayoung Song^†^, Hyen Joo Park, Sam Sik Kang, Sang Kook Lee^*^

College of Pharmacy, Natural Products Research Institute, Seoul National University, Seoul 08826, Republic of Korea

^†^These two authors contributed equally to this work.

correspondence to:

[sklee61@snu.ac.kr](mailto:sklee61@snu.ac.kr)

1. Isolation of Glabralactone S1
2. NMR Spectra of Glabralactone S2
3. HPLC analysis of Glabralactone S3

1 Isolation of Glabralactone

The dried and crushed roots of the plant (*Angelica sinensis*) were extracted 3 times by refluxing with 15 liters each of hexane, then ethyl acetate, acetone, and methanol for 5 h (for each extraction). Each solution was concentrated under reduced pressure to give the corresponding extract: hexane extract, ethyl acetate extract, acetone extract, and methanol extract. The hexane extract was further treated with hexane at room temperature and divided into soluble and insoluble parts. The insoluble part was added to the ethyl acetate extract, and then it was isolated by column chromatography on silica gel with a mixture of hexane and ethyl acetate. Further purification by column chromatography with a mixture of hexane and ethyl acetate and subsequent recrystallization from hexane-ethyl acetate yielded glabralactone as white needles.

2 NMR Spectra of Glabralactone

Routine ^1^H NMR spectra were recorded on Bruker Unity 400 MHz spectrometers at ambient temperature unless otherwise stated. Spectra were processed using MestReNova 6.0 using the automatic phasing and polynomial baseline correction capabilities. ^1^H Resonances are referenced to solvent residual peaks for CDCl_3_ (7.26 ppm). Routine ^13^C NMR spectra were recorded on Bruker Unity 400 MHz spectrometers with protons fully decoupled. ^13^C Resonances are reported in ppm relative to solvent residual peaks for CDCl_3_ (77.16 ppm).

**^1^H NMR Spectrum of glabralactone (400 MHz, CDCl_3_)**


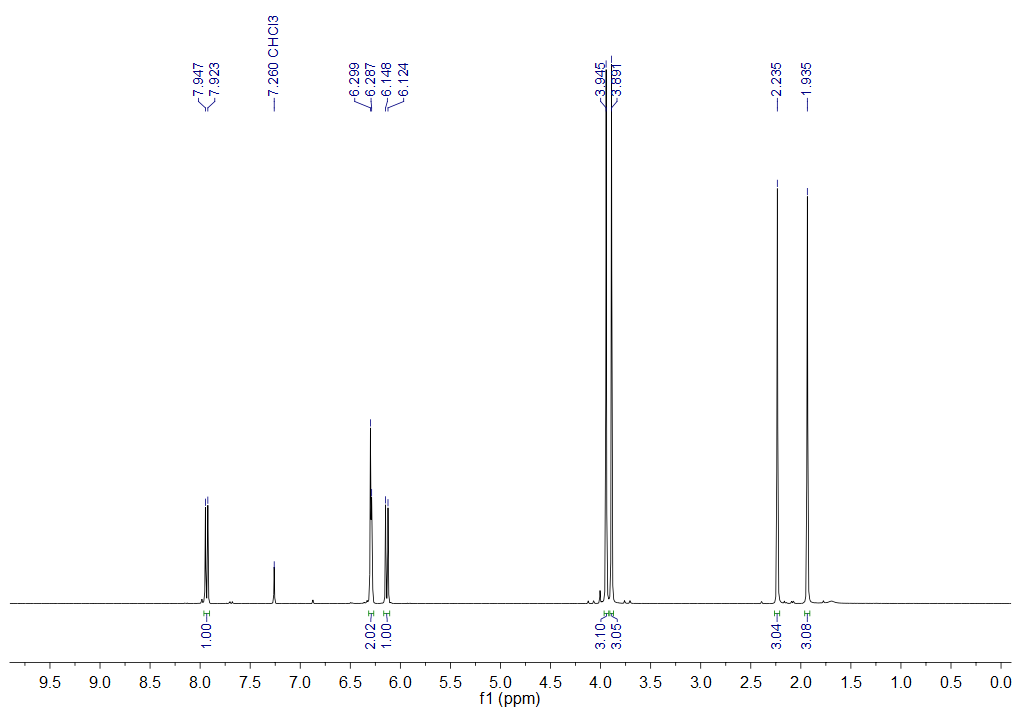


**^13^C NMR Spectrum of glabralactone (100 MHz, CDCl_3_)**


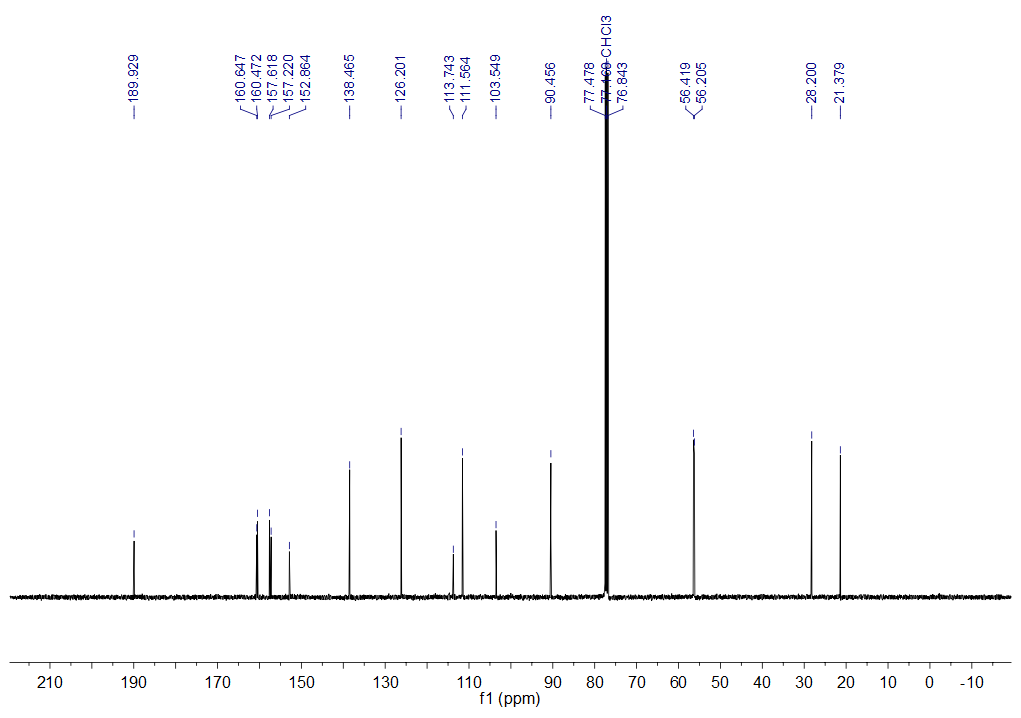


3 HPLC analysis of Glabralactone

High-performance liquid chromatography (HPLC) analysis was performed using a Waters HPLC system (Waters Alliance 2965 Separation module, Waters, MA, USA) equipped with a PDA detector (Waters 2995 Photodiode-Array detector) and a reversed-phase COSMOSIL C18 column (4.6 × 250 mm, 5 μm).

Mobile phase: **A** - H_2_O with 0.1% trifluoroacetic acid; **B** - MeCN with 0.075% trifluoroacetic acid

Flow rate: 1 mL/min

Elution: 20% of **A**/**B** to 80% of **A**/**B** over 30 min

Wavelength: 254 nm

**Purity of glabralactone: 96%**


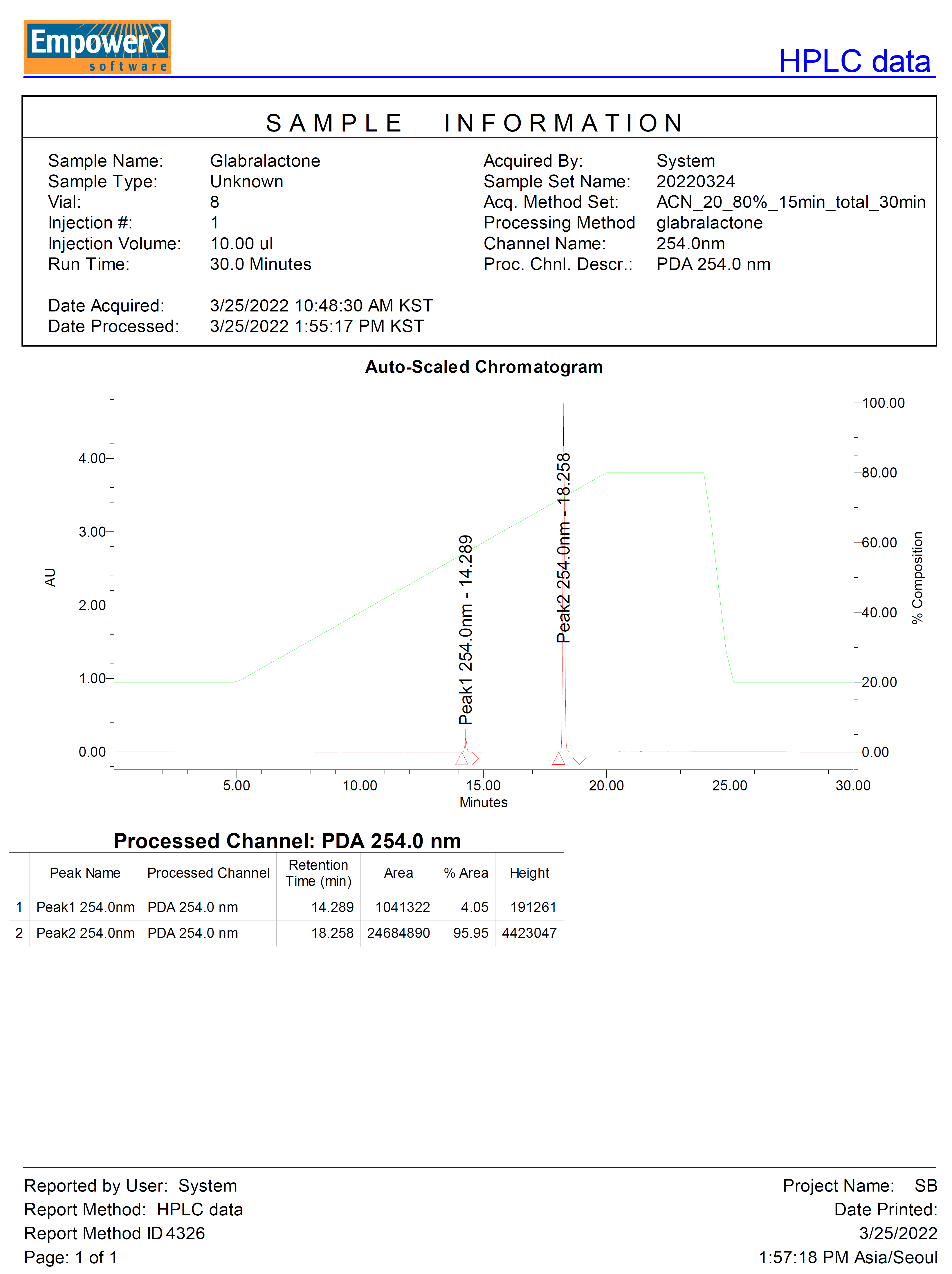

Supplement: Supplementary Materials — The Supplementary Material is available at https://www.hindawi.com/journals/mi/, which includes isolation of glabralactone, copies of NMR spectra, and HPLC trace (PDF). [file 5985255.f1.docx]
